# Proteomics and disease network associations evaluation of environmentally relevant Bisphenol A concentrations in a human 3D neural stem cell model

**DOI:** 10.3389/fcell.2023.1236243

**Published:** 2023-08-16

**Authors:** Alex Horánszky, Bachuki Shashikadze, Radwa Elkhateib, Salvo Danilo Lombardo, Federica Lamberto, Melinda Zana, Jörg Menche, Thomas Fröhlich, András Dinnyés

**Affiliations:** ^1^ BioTalentum Ltd., Gödöllő, Hungary; ^2^ Department of Physiology and Animal Health, Institute of Physiology and Animal Nutrition, Hungarian University of Agriculture and Life Sciences, Gödöllő, Hungary; ^3^ Laboratory for Functional Genome Analysis (LAFUGA), Gene Center, LMU Munich, Munich, Germany; ^4^ Max Perutz Labs, Vienna Biocenter Campus (VBC), Vienna, Austria; ^5^ Department of Structural and Computational Biology, Center for Molecular Biology, University of Vienna, Vienna, Austria; ^6^ CeMM Research Center for Molecular Medicine of the Austrian Academy of Sciences, Vienna, Austria; ^7^ Faculty of Mathematics, University of Vienna, Vienna, Austria; ^8^ Department of Cell Biology and Molecular Medicine, University of Szeged, Szeged, Hungary

**Keywords:** bisphenol A, neural stem cells, human induced pluripotent stem cells, DOHaD, new approach methodology

## Abstract

Bisphenol A (BPA) exposure is associated with a plethora of neurodevelopmental abnormalities and brain disorders. Previous studies have demonstrated BPA-induced perturbations to critical neural stem cell (NSC) characteristics, such as proliferation and differentiation, although the underlying molecular mechanisms remain under debate. The present study evaluated the effects of a repeated-dose exposure of environmentally relevant BPA concentrations during the *in vitro* 3D neural induction of human induced pluripotent stem cells (hiPSCs), emulating a chronic exposure scenario. Firstly, we demonstrated that our model is suitable for NSC differentiation during the early stages of embryonic brain development. Our morphological image analysis showed that BPA exposure at 0.01, 0.1 and 1 µM decreased the average spheroid size by day 21 (D21) of the neural induction, while no effect on cell viability was detected. No alteration to the rate of the neural induction was observed based on the expression of key neural lineage and neuroectodermal transcripts. Quantitative proteomics at D21 revealed several differentially abundant proteins across all BPA-treated groups with important functions in NSC proliferation and maintenance (e.g., FABP7, GPC4, GAP43, Wnt-8B, TPPP3). Additionally, a network analysis demonstrated alterations to the glycolytic pathway, potentially implicating BPA-induced changes to glycolytic signalling in NSC proliferation impairments, as well as the pathophysiology of brain disorders including intellectual disability, autism spectrum disorders, and amyotrophic lateral sclerosis (ALS). This study enhances the current understanding of BPA-related NSC aberrations based mostly on acute, often high dose exposures of rodent *in vivo* and *in vitro* models and human GWAS data in a novel human 3D cell-based model with real-life scenario relevant prolonged and low-level exposures, offering further mechanistic insights into the ramifications of BPA exposure on the developing human brain and consequently, later life neurological disorders.

## 1 Introduction

The Developmental Origins of Health and Disease (DOHaD) concept relates early-life environmental conditions, including chemical exposures, to metabolic and health status in adult life. These exposures are associated with the onset of non-communicable diseases throughout life, including brain disorders (Haugen et al., 2015; Fleming et al., 2018). BPA is an endocrine disrupting compound (EDC) commonly utilized as a chemical precursor in the industrial manufacturing of epoxy resins, polycarbonate plastics, food and drink packaging and many other items found in daily consumer life (Vandenberg et al., 2007; Porras et al., 2014). The use of BPA-containing products is widespread, which is concerning due to their capacity to depolymerize and leach into the environment, for example, into food and drink stored in cans and plastic containers (Brede et al., 2003; Munguía-López et al., 2005; Le et al., 2008). BPA has also contaminated the environment in the air, water, soil and dust, and therefore potential routes of human uptake include inhalation and dermal absorption, as well as dietary ingestion from contaminated food (Hartle et al., 2022).

It has been well documented that BPA exposure is associated with altered cognitive function and behaviour, as well as neurodevelopmental disorders (NDDs) and neurodegenerative diseases (NDs), such as Alzheimer’s disease (AD), autism spectrum disorders (ASD), and schizophrenia (Brown, 2009; Rebolledo-Solleiro et al., 2021). BPA has been identified in human blood, fetal serum, and breast milk (Rebolledo-Solleiro et al., 2021) and its lipophilic properties enable its permeation across cell membranes, placenta, and fetal blood-brain-barrier (BBB) (Ikezuki et al., 2002; Balakrishnan et al., 2010) allowing it to affect embryonic and fetal stages of brain development (O’Shaughnessy et al., 2021).

Estimations suggest that human intake of BPA is approximately 1-5 ug/kg/day (Murata and Kang, 2018), and due to its abundance in the environment, many people are subjected to a chronic exposure (Welch and Mulligan, 2022). Importantly, previous studies demonstrate that BPA doses in the ng/kg/day range, significantly lower than estimated daily human intake, induce aberrations to brain development in animal models (Negri-Cesi, 2015; Welch and Mulligan, 2022). Exposure of the vulnerable fetal brain to environmental chemicals could cause disruption to early developmental processes; from neurulation, neuronal proliferation, differentiation, and migration, through to synaptogenesis, dendritic outgrowth and myelination (Schneider et al., 2003; Hornung et al., 2009; Guo et al., 2013; Ling et al., 2016; Huang et al., 2019; Chesnut et al., 2021; Kalloo et al., 2021). Primary neurulation is the earliest stage of fetal central nervous system (CNS) development, where the neuroectoderm is derived from the ectodermal germ layer and NSCs first appear in the developing brain (Nikolopoulou et al., 2017; Leibovitz et al., 2022).

Initially, brain growth is facilitated by NSC expansion via symmetric cell divisions, followed by asymmetric NSC divisions, producing an NSC and an intermediate neuronal progenitor (NPC) in the early stages of neurogenesis. Even though NPCs have a limited proliferative capacity compared to NSCs as they enter a “primed” state for neuronal differentiation, their division is maintained throughout neurogenesis, providing a renewing pool of progenitors for further neuronal/glial specification (Bergström and Forsberg-Nilsson, 2012; Suzuki et al., 2021). In attempts to clarify its effects on the fetal stages of brain development, several studies have explored the impact of BPA exposure on NSC populations. Both *in vitro* and *in vivo* investigations have described conflicting reports showing both increases and decreases to NSC proliferation depending on BPA concentration and duration of exposure. Notably, most *in vitro* studies used BPA treatments ranging from 24 h to 7 days, modelling the effects of acute exposure on already established NSCs, (Kim et al., 2007; Tiwari et al., 2015; Agarwal et al., 2016; Huang et al., 2019; Dong et al., 2021; Gill and Kumara, 2021), therefore the effects of BPA exposure on NSCs over longer time periods *in vitro* remain unexplored. The importance of acute toxicity testing of BPA for protecting the environment and public health cannot be understated. However, for an enhanced risk assessment of BPA’s effects on neural development and its potential contributions to NDs and NDDs, improved *in vitro* models that can determine the long-term effects of BPA after repeated exposures are needed. Importantly, in chronic exposure conditions with sub-cytotoxic concentrations, cellular responses to BPA could be much more complex, especially when considering that BPA is known to exert its intracellular effects via the interference of several receptors including nuclear receptors and noncanonical steroid hormone receptors (Acconcia et al., 2015).

A tightly regulated balance between NSC proliferation and differentiation is vital for neurogenesis and proper brain functionality, and disruptions to this balance are frequently observed in NDDs (Chen et al., 2014; Ernst, 2016; Casas Gimeno et al., 2022). Several studies have already demonstrated that BPA disturbs these processes, at least in part, via disruptions to critical signalling pathways in NSC development, such as Wnt/B-Catenin, ERRα, TGF-β, JNK, CREB and p53 pathways (Liu et al., 2015; Liu et al., 2013; Tiwari et al., 2015; 2016; Dong et al., 2021; Welch and Mulligan, 2022). Despite the wide interest and extensive studies, the mechanisms underlying BPA-induced alterations to NSCs, and their possible associations to aberrant brain development, NDDs and NDs are still under debate and require further elucidation. Quantitative proteomic approaches utilize state-of-the-art technologies that can be applied to assess cellular proteome alterations after exposure with environmental chemicals, clarifying mechanisms of toxicity and improving chemical risk assessments (Gao et al., 2009). Proteomic-based methods are advantageous compared to transcriptomic methods, as they directly quantify protein levels rather than using transcript levels as a proxy. Therefore, proteomics can be used for a deeper understanding of the effects of BPA exposure directly on the protein expression levels of genes of interest.

HiPSCs are derived from reprogrammed somatic cells, and are characterized by their capabilities of self-renewal, proliferation, and differentiation into all three embryonic germ layers (Shi et al., 2017). The neuronal differentiation of hiPSCs provides a valuable platform for further study of the effects of environmental chemicals on the developmental processes of the human CNS, bridging the gap between data from animal models and human studies (Xie et al., 2020). 3D hiPSC-derived models are particularly promising; compared to 2D cell culture systems, 3D cultures more closely replicate complex human tissues of interest with regards to cell signalling, differentiation capacity and tissue organization, while they also display more realistic responses to environmental chemicals (Kobolak et al., 2020; Wang et al., 2023). The neural induction of hiPSCs to NSCs can be used as a representative *in vitro* model of the critical neurulation stage of embryonic brain development, where NSCs are first derived in the developing brain (Chambers et al., 2009; Galiakberova and Dashinimaev, 2020).

In the present study, for the first time, we performed a repeated-dose BPA exposure for 21 days from the initiation of the *in vitro* 3D neural induction of hiPSCs. Our human cell-based system facilitated further investigation of BPA-induced perturbations during the earliest stages of embryonic brain development over a longer-term exposure than previous *in vitro* studies, supporting previous data obtained from animal studies in a human relevant model. The expression of key neuroectodermal and neural lineage markers demonstrated that our system provides a robust model for NSC differentiation to investigate the effects of environmentally relevant BPA concentrations. This allowed us to support current literature and elucidate BPA-induced aberrations to NSC properties, such as inhibited proliferation. Additionally, we utilized a quantitative proteomics approach alongside a disease network analysis to investigate proteome-wide alterations in BPA-treated NSCs, uncovering novel BPA-induced molecular changes in NSCs that could be implicated in altered NSC properties during development and the pathophysiology of certain brain disorders.

## 2 Materials and methods

### 2.1 Chemicals and plasticware

Chemicals were purchased from Sigma-Aldrich (St Louis, MO, United States). Plasticware and reagents for cell culture were purchased from Thermo Fisher Scientific Inc (Waltham, MA, United States), unless stated otherwise.

### 2.2 hiPSC culture

The SBAD2 hiPSC line, derived from healthy adult dermal fibroblast (NHDF-Ad) cells (Lonza, Cat#: CC-2511, 51 years old Caucasian male) reprogrammed using non-integrative Sendai virus transduction, was used in this study. Cells were maintained *in vitro* in a humidified atmosphere that contained 5% CO_2_ at 37°C. BD Matrigel™ matrix (BD Biosciences) was used for plate coating and cells were maintained with mTeSR™1 medium (Stem Cell Technologies). EDTA (0.02% Versene, Cat#: BE17-711E, Lonza) was used for passaging cells every 5–7 days as described in the manufacturer’s protocol. The Venor®GeM-Advance (Minerva Biolabs) *Mycoplasma* Detection Kit was used for routine *mycoplasma* screening, as described in the manufacturer’s instructions. Representative characterization of the SBAD2 hiPSCs was performed previously, demonstrating normal stem cell characteristics including colony morphology, karyotype, and expression of pluripotency markers (Snijders et al., 2021; Fehér et al., 2022).

### 2.3 3D neural induction

The *in vitro* differentiation of hiPSCs to NSCs was carried out using the dual SMAD inhibition method (Chambers et al., 2009; Shi et al., 2012) to induce neuroectoderm formation. Upon reaching 90% confluence, the neural induction was initiated by replacing mTeSR™1 medium with neural induction medium (NIM; Neurobasal medium: DMEM/F12, supplemented with 1x N2, 2x B27, 100 µM β-mercaptoethanol, 2 mM glutamine, 1x non-essential amino acid (NEAA), 5 μg/mL insulin) supplemented with 200 nM LDN-193189 HCL (Selleck Chemicals, cat# S7507), 10µM SB431542, and 5 ng/mL basic fibroblast growth factor (bFGF). The next day, hiPSCs were dissociated with Accutase^®^ solution and seeded as single cells (10,000 cells/well) onto Prime Surface 96well V plates in 200 µL NIM for spheroid formation. NIM was replaced every third day with a 75% media change, to minimize spheroid disturbance.

### 2.4 BPA-treated 3D neural induction

BPA stock solution was prepared by dissolving the compound in DMSO to a final concentration of 100 mM. BPA was further diluted to appropriate experimental concentrations using NIM. Repeated doses of 0.01, 0.1 and 1 µM BPA were administered to spheroids at each medium change for 3 weeks following the initiation of the 3D neural induction (Figure 3C). Media change and BPA treatments were performed every third day to minimize spheroid disturbance. 75% of spent medium was exchanged with NIM containing concentrations of BPA that yielded the specified treatment concentrations. NIM supplemented with 0.1% DMSO was used as vehicle control throughout the experiment.

### 2.5 Immunocytochemical staining

3D NSC spheroids were fixed with 4% PFA in 0.1 mol/L phosphate buffer for 1 h at RT and subsequently washed 3 times with phosphate buffered saline (PBS). Spheroids were permeabilized with 0.2% TritonX-100 in PBS and blocked in 3% BSA in PBS at RT for 1 h. Spheroids were incubated with primary antibodies (Supplementary Table S1) overnight at 4°C. Spheroids were then washed 3 times in PBS before their incubation with isotype-specific secondary antibodies diluted in 3% BSA in PBS for 1 h at RT. Spheroids were washed 3 times in PBS and mounted on Superfrost™ Ultra Plus Adhesion Slides (Thermo Fisher Scientific) using ProLong™ Diamond Antifade Mountant with DAPI for nuclear labelling. Image acquisition was undertaken using a BX-41 epifluorescent microscope (objectives: ×20 0.50 NA; 40 × 0.75 NA; Olympus) fitted with a DP-74 digital camera and Cellsens software (V1.18; Olympus).

### 2.6 RT-qPCR

At each sampling timepoint, 16 spheroids were combined, and 3 independent experiments were performed (*n* = 3). RNA isolation was performed using either the RNeasy Plus Micro Kit (Qiagen), or the RNeasy Plus Mini Kit (Qiagen). The Maxima First Strand cDNA Synthesis Kit for RT-qPCR with dsDNase (Thermo Fisher Scientific) was used for the reverse transcription using 1500 ng of the isolated RNA, as described in the manufacturer’s protocol. Primer3 software was used to design gene-specific primers (Supplementary Table S2) and GAPDH was used as a reference gene. 5ng cDNA template was used for each qPCR reaction in addition to 50% SYBR Green JumpStart Taq ReadyMix and 400 nM of each primer, to a final volume of 15 µL. The QIAgility liquid handling robot and Rotor-Gene Q cycler (Qiagen) were used for experimental setup and qPCR reaction, respectively. The qPCR cycling protocol included 3 min at 94°C for denaturation and a subsequent 40 cycles of 5 s at 95°C, 15s at 60°C, and 30 s at 72°C. Primer specificity was confirmed via melting curve analysis. Fetal Brain RNA (Takara Bio, Cat# 636526) and human cortical RNA (Takara Bio, Cat# 636561) were used for normalization. The ddCT method (Livak and Schmittgen, 2001) was used to analyze data obtained from three technical replicates for each gene.

### 2.7 Cell viability and cytotoxicity assays

#### 2.7.1 ATP viability assay

NSC spheroids were treated with increasing concentrations of BPA for 72 h (Figure 3A) to produce dose-response curves. In each experimental plate, 3 technical replicates were used for each concentration, and data were obtained from 3 independent assays (*n* = 3). Vehicle control was used as NIM supplemented with 0.1% DMSO. Paraquat was used as a positive neurotoxic control. CellTiter-Glo^®^ 3D Cell Viability Assay (Promega) was used to perform the ATP viability assay, as described in the manufacturer’s instructions. After BPA treatment, 100 µL CellTiter-Glo^®^ 3D Reagent was added to the NSC spheroids for 1 h at RT. The luminescent signal was then recorded using a Thermo VarioScan Flash plate reader (Thermo Fisher Scientific).

#### 2.7.2 LDH cytotoxicity assay

Spheroids were subjected to a repeated-dose exposure using nanomolar concentrations of BPA for 21 days during the neural induction process, as previously described (Figure 3C). For each BPA concentration and vehicle control, treated NIM was removed from triplicate wells at D14 and D21 of the BPA-treated neural induction to analyze LDH release. Cytotoxicity was calculated on day 14 and day 21 of BPA treatment using negative controls on the corresponding days. Three independent experiments were performed (*n* = 3). The CyQUANT™ LDH Cytotoxicity Assay (Thermo Fisher Scientific) was used to measure LDH levels from the collected medium to quantify cytotoxicity, as described in the manufacturer’s protocol. The colorimetric signal was measured using a Thermo VarioScan Flash plate reader.

### 2.8 Spheroid size analysis

Vehicle-control, and BPA-treated NSC spheroids were imaged using the ×4 objective (0.1NA) of the Olympus IX71 microscope and DP21 camera (DP21). The captured images were analyzed via surface area measurements using the CellSens Dimension software (V1.11). Each value represents the average of 4 independent experiments (*n* = 4) where 8 spheroids at each concentration were measured.

### 2.9 Analysis of ROS and mitochondrial levels

NSC spheroids at D21 of the BPA treatment protocol were treated with 5 µM CellROX™ Deep Red (Invitrogen) for 1 h at 37°C, or 100 nM MitoTracker Deep Red 633 (Invitrogen) for 30 m at 37°C, for the assessment of reactive oxygen species (ROS) and mitochondrial levels, respectively (ex/em; 630/650 nm). Spheroids were then fixed with 4% PFA in 0.1 mol/L phosphate buffer for 1 h at RT. The IncuCyte^®^ Live Cell Analysis System (Sartorius, United States), was then used for image capture and quantification of the fluorescence signal (exposure time: 150 ms). Assessed spheroids were obtained from 3 independent experiments (*n* = 3).

### 2.10 Quantitative proteomics

#### 2.10.1 Sample preparation

Cells were lysed in 8 M urea/0.5 M NH4HCO3 by ultrasonication (18 cycles of 10 s) using a Sonopuls HD3200 (Bandelin, Berlin, Germany). Proteins were quantified using Pierce 660 nm Protein Assay (Thermo Fisher Scientific, Rockford, IL, United States). 20 µg of protein was reduced with 4 mM dithiothreitol (DTT) and 2 mM tris(2-carboxyethyl) phosphine (TCEP) at 56°C for 30 min and alkylated with 8 mM iodoacetamide (IAA) at room temperature in the dark. DTT was added to a final concentration of 10 mM to quench residual IAA during 15 min incubation in the dark. Proteins were digested with modified porcine trypsin (1:50 enzyme/protein ration, Promega) for 16 h at 37°C.

#### 2.10.2 Nano-liquid chromatography (LC)–tandem mass spectrometry (MS/MS) analysis and statistics

1 μg of the digest was injected on an UltiMate 3000 nano-LC system coupled online to a Q-Exactive HF-X instrument (Thermo Fisher Scientific). Peptides were first transferred to a PepMap 100 C18 trap column (100 µm × 2 cm, 5 µM particles, Thermo Fisher Scientific) and separated on an analytical column (PepMap RSLC C18, 75 µm × 50 cm, 2 µm particles, Thermo Fisher Scientific) at 250 nL/min flow-rate with an 80-min gradient of 5%–20% of solvent B followed by a 9-min increase to 40%. Solvent A consisted of 0.1% formic acid in water and solvent B of 0.1% formic acid in acetonitrile. MS spectra were acquired using a top 15 data-dependent acquisition method on a Q Exactive HF-X mass spectrometer. The dataset has been submitted to the ProteomeXchange Consortium via the PRIDE partner repository (Perez-Riverol et al., 2022) with the dataset identifier PXD042045. Raw file processing was performed using MaxQuant (Tyanova et al., 2016) using the human SwissProt reference proteome downloaded in October 2022. All statistical analyses and data visualization were performed in R using custom scripts. Proteins with at least two peptides detected in at least three samples of each condition were tested for differential abundance using the MS-EmpiRe (Ammar et al., 2019) algorithm as described previously (Flenkenthaler et al., 2021). To handle missing values for peptides with measurements in all replicates of one condition and insufficient measurements in the other condition, data imputation with random numbers from the normal distribution (downshift 1.8, width 0.3) was performed. Proteins with a Benjamini–Hochberg corrected *p*-value ≤0.05 and fold-change ≥1.3 were regarded as significantly altered. For hierarchical clustering ComplexHeatmap R package (Gu et al., 2016) was used. The k-means algorithm was used for partitioning the heatmap into homogeneous regions. Over-representation analysis was performed using the WebGestaltR package (Liao et al., 2019) and the functional category ‘GO Biological Process nonRedundant’. The false discovery rate was controlled using the Benjamini–Hochberg method.

### 2.11 Statistical analysis

Data is presented with the mean ± standard error of the mean (SEM). All data, excluding proteomics analysis, was analyzed using Prism 7 (Graphpad Software, CA, United States) software. Statistical significance was determined using One-way ANOVA, or two-way ANOVA and Dunnett’s post hoc test, where applicable. A *p*-value of <0.05 was considered significant.

### 2.12 Network analysis

#### 2.12.1 Human protein-protein interaction (PPI) network construction and analysis

The human PPI network was built from publicly available resources (Menche et al., 2015; Alanis-Lobato et al., 2017; Luck et al., 2020), resulting in 18,816 proteins and 478,353 physical interactions.

Significantly changed proteins were mapped onto the human PPI network, and their connectivity was calculated by computing a z-score of the largest connected component for each group of proteins compared to 10,000 randomly selected protein sets of the same size. For each condition, we considered all differentially abundant proteins, as well as subdividing into up- and down-regulated proteins.

#### 2.12.2 Enrichment analysis

Differentially abundant proteins and their respective connected core were biologically characterized by performing an enrichment analysis for the three main branches of the gene ontology (GO) (Ashburner et al., 2000): biological processes (BP), molecular functions (MF), and cellular components (CC), and for KEGG pathway (Kanehisa and Goto, 2000) using GSEAPY (Fang et al., 2023).

#### 2.12.3 Disease predictions

Diseases-gene associations (GDA) were retrieved from DisGeNet (Piñero et al., 2015) which represents the largest publicly available collections of genes and variants associated with human diseases, including expert-curated associations from GWAS catalogues, animal models and scientific literature. Depending on the accuracy of the type of information, each gene-disease association is attributed with a GDA score that ranges from 0 to 1. We selected only associations with a GDA score >0.3, retrieving information for 11,099 diseases. The relationship between each set of differentially abundant proteins (s1) and set of disease proteins (s2) was then computed in two different ways: 1) by calculating their Jaccard index (intersection (s1,s2)/union(s1,s2)), and by network proximity of the two sets (Guney et al., 2016). Network proximity computes the closest distance between two sets of proteins in a network and by comparing it against 10,000 random sets of similar topological features. In this way, we considered and corrected for interactome biases such as the heavy-tail degree distribution and the discretization of other common network distances like the shortest path.

The closest path between the differentially abundant proteins and the disease genes was computed by considering both the shortest path and the presence of hubs in the direct proximity of the two gene sets (source code available at: github.com/superlsd/NetBPAbrain).

#### 2.12.4 ALS candidate genes validation

Gene expression count data from human post-mortem spinal cords were downloaded from zenodo platform (Humphrey et al., 2023). The normalized Transcripts Per Kilobase Million (TPM) values of the two most relevant regions for disease progression (cervical and lumbar) were used for further statistical analyses. To compare the median expression of the connecting genes between ALS and BPA-downregulated genes in both ALS patients and controls, we performed a two-tailed *t*-test.

### 2.13 Ethical approval

The ethical licence was issued by the Scientific and Research Ethics Committee of the Hungarian Health Science Council for “Production of induced pluripotent stem cells (IPS) from human somatic samples” with the following ID No.: IV/3935- 1/2021/EKU in May 2021.

## 3 Results

### 3.1 The characterization of hiPSC-derived NSCs confirms the 3D neural induction as a robust *in vitro* model for NSC differentiation

**FIGURE 1:**
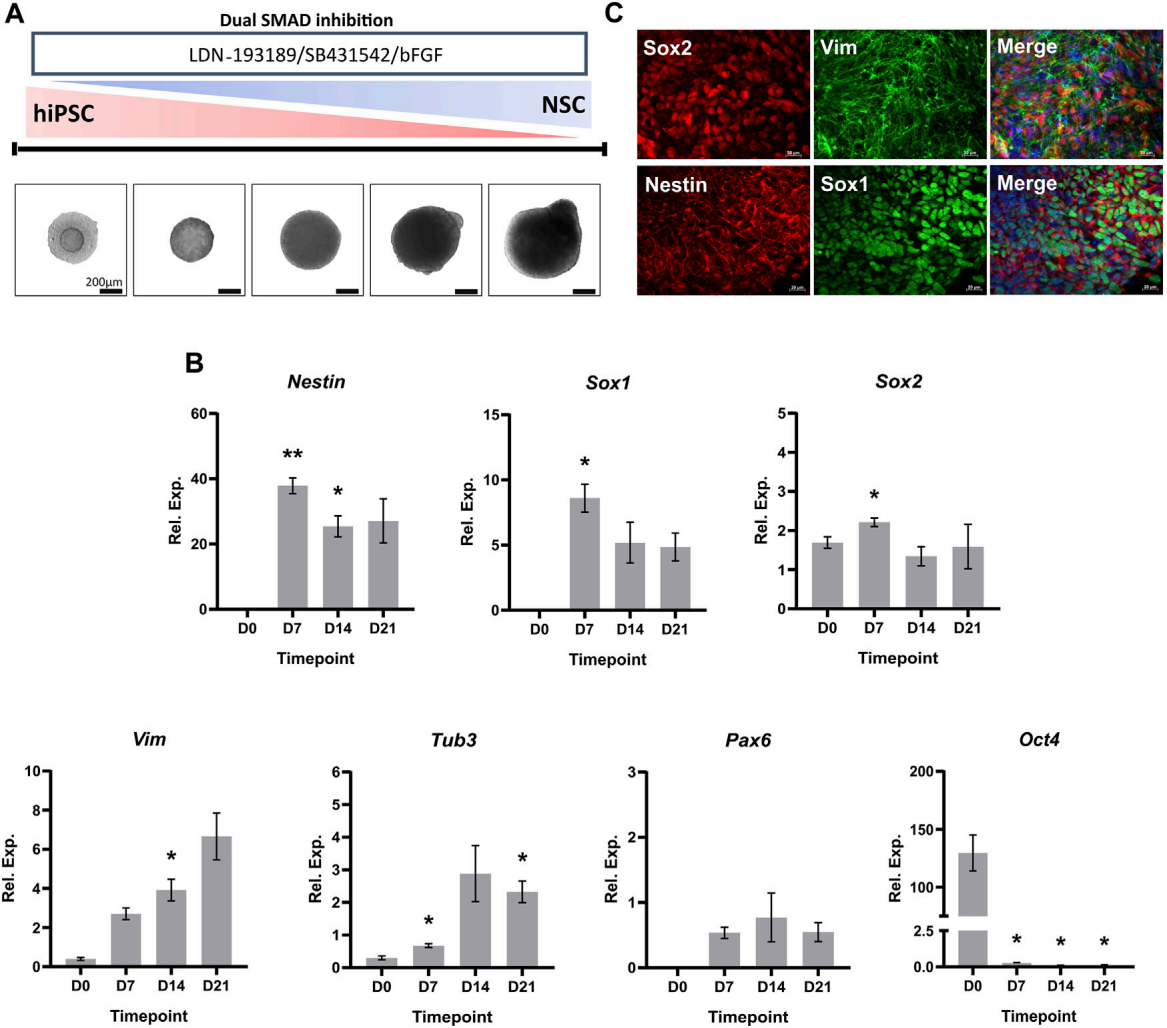
Characterization of hiPSC-derived NSCs. **(A)** Schematic overview and phase contrast images of the 3D *in vitro* neural induction of hiPSCs to NSCs. Scale bar, 200 µm. **(B)** Real-time qPCR measurements of the 3D neural induction of hiPSCs. Graphs represent normalized relative expression values. GAPDH was used as a reference gene and data was normalized using Takara human cortical RNA. The results shown are from 3 independent experiments (*n* = 3). One-way ANOVA with Dunnett’s post hoc test was used to determine significance (adjusted *p*-value * *p* < 0.05, ** *p* < 0.01). ± SEM are displayed. **(C)** Neuroectodermal and NSC markers were stained in 3D culture (×40 magnification). The used fluorophores were Alexa 488 (green) and Alexa 594 (red). Nuclei were counterstained with DAPI (blue). Scale bar, 50 µm.

We performed a characterization of the hiPSC-derived NSCs to assess the suitability of the 3D neural induction as an *in vitro* model for NSC differentiation. Upon initiation of the 21-day induction process via dual-SMAD inhibition, hiPSCs formed compact spheroids within 48 h. Furthermore, brightfield images demonstrated a consistent growth of spheroid size throughout the differentiation (Figure 1A). Next, we investigated the expression levels of key transcripts associated with NSCs throughout the neural induction, via RT-qPCR (Figure 1B). There was a significant increase in the relative expression of classical neuroectodermal markers Nestin and Sox1, and marker of NSC multipotency Sox2, by D7 of the differentiation. Over the course of the induction, progressive increases in the mRNA levels of genes critical to the neural lineage, such as Tubulin beta-3 chain (Tub3) and Vimentin, were accompanied by a marked decrease in the expression of pluripotency marker Oct-4 by D7. The expression of Nestin, Sox1, Sox2 and Vimentin observed in Figure 1B was confirmed on a protein level using ICC detection of NSC spheroids at D21 of the neural induction (×40 magnification) (Figure 1C, Supplementary Figure S3).

**FIGURE 2:**
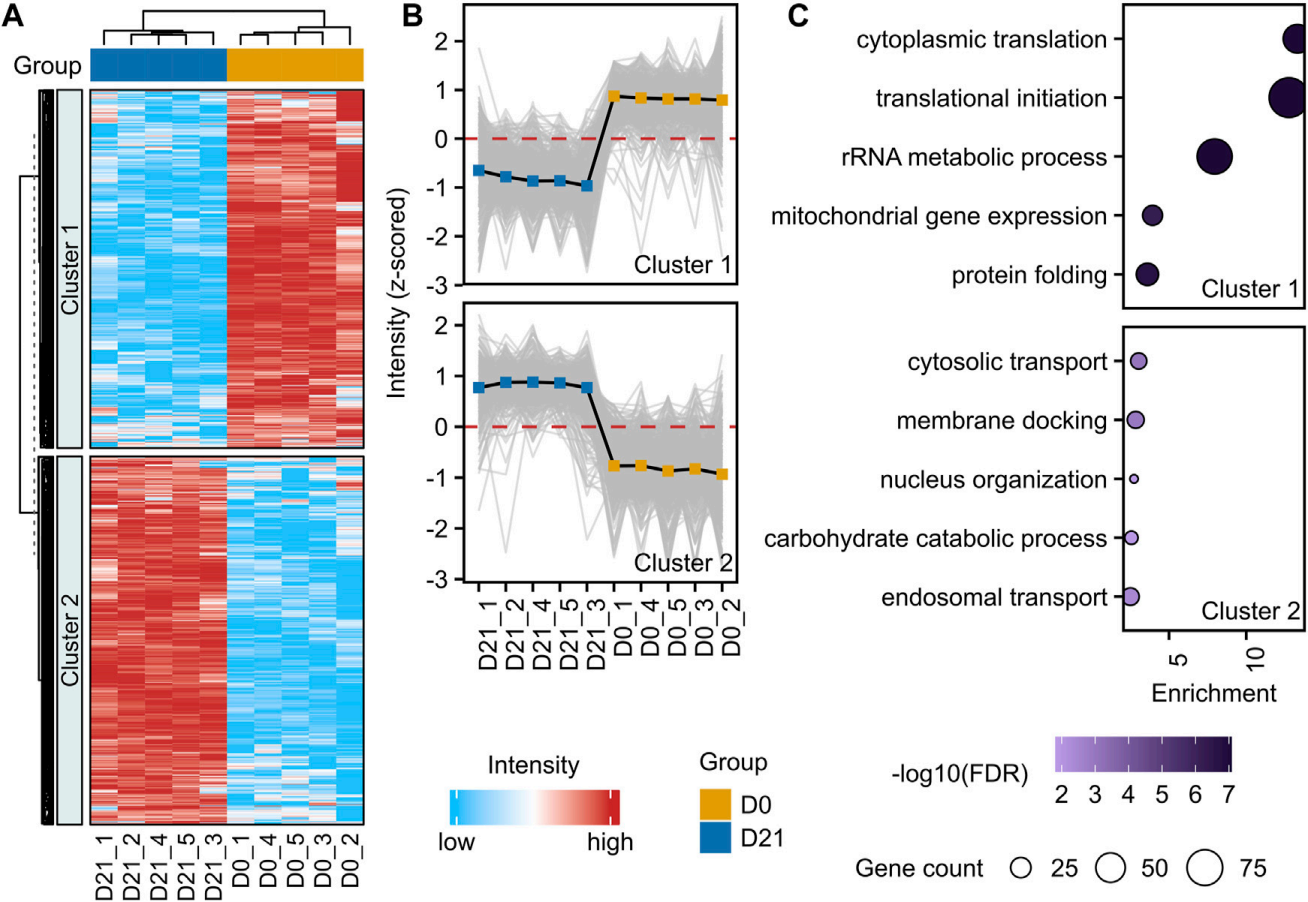
Proteome alteration upon neural induction of hiPSCs. **(A)** Unsupervised hierarchical clustering of significantly changed proteins (adjusted *p*-value <0.05, fold-change >1.3) between D0 and D21 (*n* = 5). Standardized protein abundance values are shown in red and light blue for high and low abundance, respectively. K-means algorithm (*k* = 2) was used to partition heatmap rows into homogenous regions. **(B)** Profile plots with mean values (solid black line) of two clusters demonstrating distinct protein alterations between D0 (hiPSC) and D21 (NSC). **(C)** Enrichment analysis of proteins from each cluster. The size of the bubble indicates the corresponding number of differentially abundant proteins (referred to as gene count in the figure), and the color of the bubble indicates the significance of the enrichment. Enrichment shows the magnitude of over-representation.

For a deeper characterization of the hiPSC-derived NSCs, we investigated proteome alterations in an unbiased and comprehensive manner. To achieve this, we performed a label-free liquid chromatography-tandem mass spectrometry analysis (LC-MS/MS) of hiPSCs (D0, *n* = 5), and NSCs (D21, *n* = 5). A total of 4733 protein groups were identified with high confidence (false-discovery rate <0.01) (Supplementary Table S4A). The neural induction of hiPSCs led to the alteration of 60% of quantified proteins (Figures 2A, B, Supplementary Table S4B). Proteins being part of cytosolic transport, membrane docking, and nucleus organization were increased, while those involved in translational initiation, cytoplasmic translation, and rRNA metabolic processes were decreased (Figure 2C, Supplementary Tables S4B, C). Proteins with the highest increases in abundance at D21 included typical NSC markers FABP7, DACH1 and DCX, and similarly other proteins typical of the neural lineage, such as MAP2, NCAM1 and MSI1 were elevated. Notably, there were increases to the abundance of neural lineage proteins Nestin, Vimentin, Sox2 and Tub3 at D21 compared to D0, in accordance with our RT-qPCR data (Figure 1B). In contrast, there was a decrease in the abundance of Yamanaka factor Oct-4, and pluripotency marker LIN28A in NSCs compared to hiPSCs.

### 3.2 Treatment with nanomolar concentrations of BPA had no effect on cellular viability during the neural induction

**FIGURE 3:**
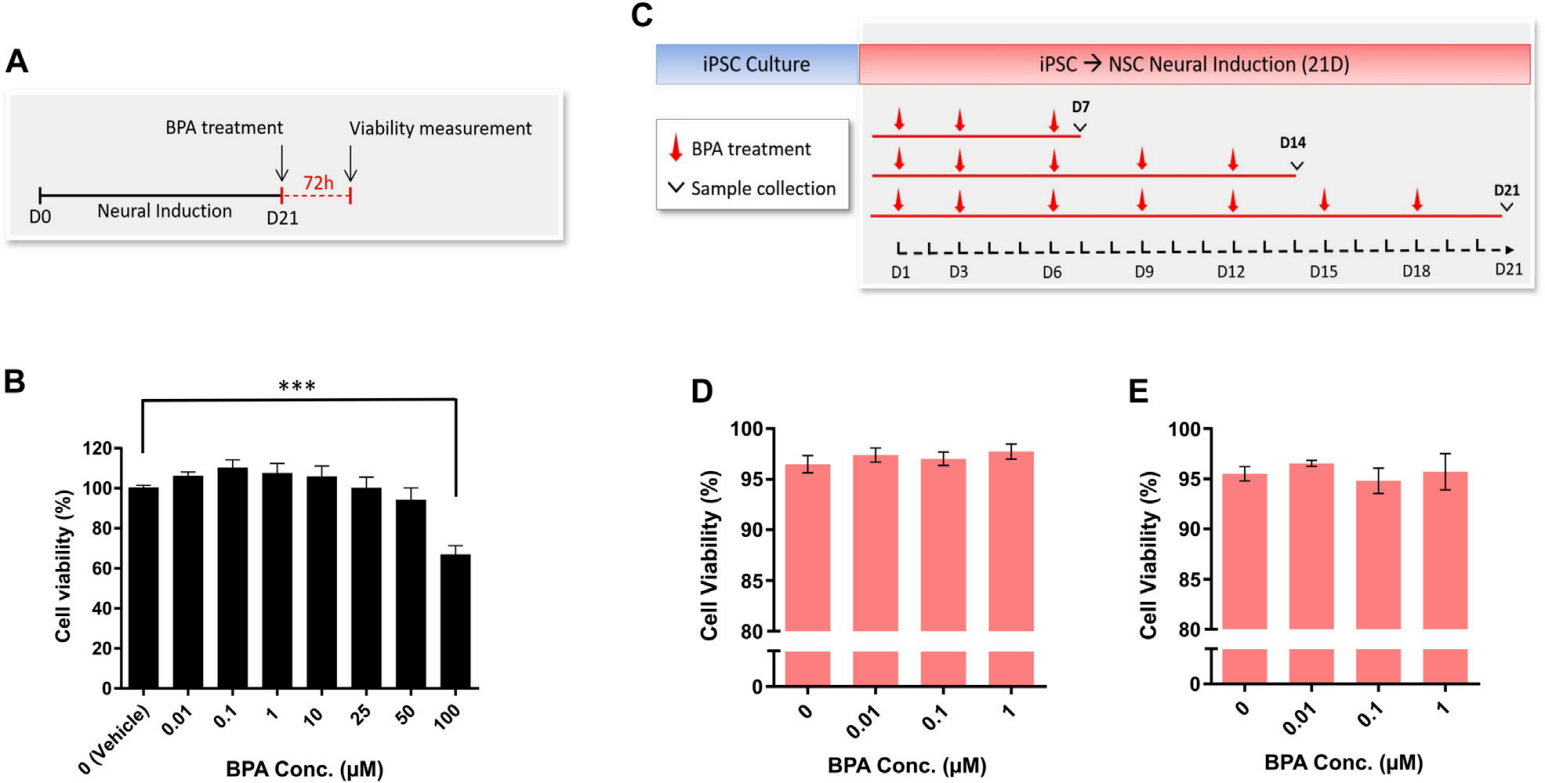
Cell viability and cytotoxicity measurement of NSC spheroids after BPA exposure. **(A)** Experimental schematic and **(B)** concentration-response representing the cell viability (%) of NSC spheroids (*n* = 3) upon 72 h BPA exposure. **(C)** Schematic outlining the repeated-dose BPA exposure during the 21-day neural induction, and LDH assay demonstrating viability (%) calculated from LDH release of NSC spheroids at **(D)** D14 (*n* = 3) and **(E)** D21 (*n* = 3) of the BPA treated neural induction. One-way ANOVA with Dunnett’s post hoc test was used to determine significance (adjusted *p*-value *** *p* < 0.001) ± SEM are displayed.

The hiPSC-derived NSCs were treated for 72 h with increasing concentrations of BPA (Figure 3A) and a cell viability assay was performed to determine sub-cytotoxic concentration levels in our setting. A single-dose BPA treatment at 100 µM significantly decreased NSC viability by 33% after 72 h. Conversely, concentrations of 0.01 µM–50 µM had no significant effect on NSC viability after 72 h BPA treatment, compared to the vehicle-treated control group (Figure 3B).

Having determined a sub-cytotoxic range of BPA concentrations that were aligned with environmentally realistic exposure levels (Ribeiro et al., 2017; 2019; Zahra et al., 2022), we performed the repeated-dose BPA exposure protocol using 0 µM (vehicle control), 0.01, 0.1 and 1 µM BPA during the neural induction of hiPSCs to NSCs (Figure 3C). Subsequently, we applied cytotoxicity assays to investigate whether a longer-term exposure to the selected concentrations would affect cell survival. At both D14 (Figure 3D) and D21 (Figure 3E) of the BPA-treated neural induction, no significant decreases in NSC viability (%) were observed in each treated group compared to the vehicle-treated control group.

### 3.3 Effects of a repeated-dose BPA exposure on NSC spheroid growth

**FIGURE 4:**
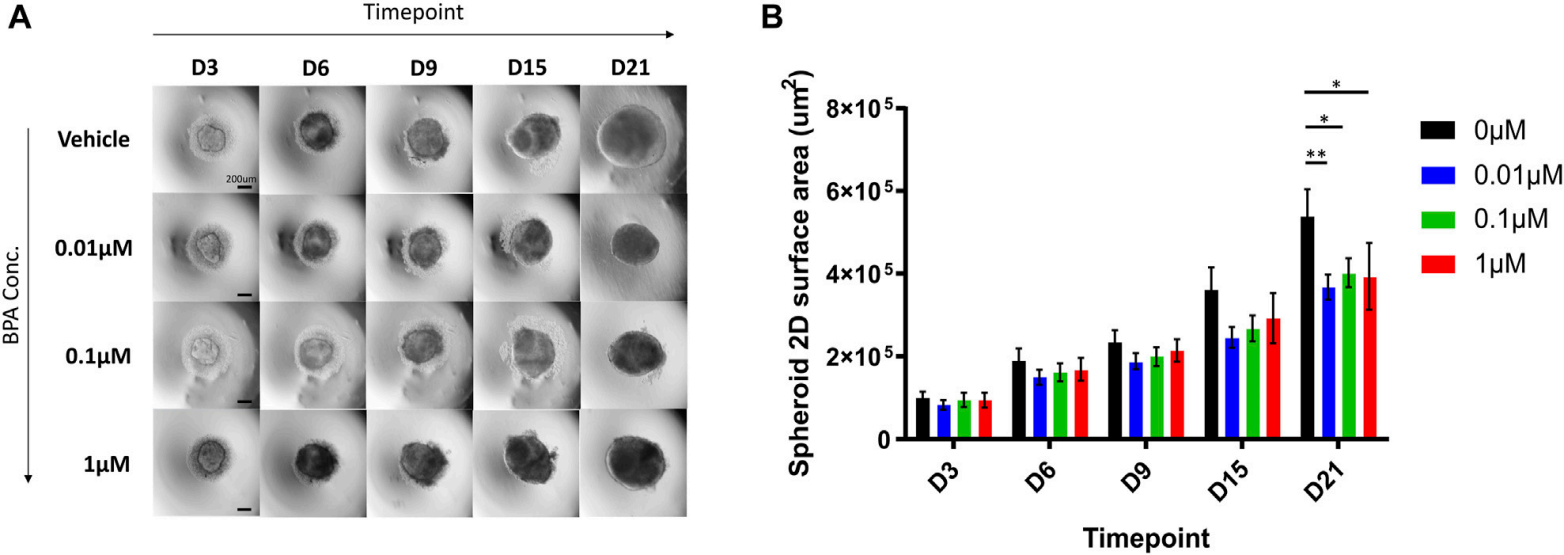
Microscopic aspect and growth curves of spheroids treated with increasing concentrations of BPA during the neural induction process. **(A)** The microscopic aspect of spheroids observed using the ×4 objective (0.1 NA) of Olympus IX71 microscope and DP21 camera. Scale bar, 200 µm. **(B)** Spheroid growth over 21 days from the beginning of the neural induction of hiPSCs with repeated dose exposure of 0.01, 0.1 and 1 µM BPA. Vehicle (0 µM) represents spheroids treated with 0.1% DMSO. Results displayed are from 4 independent experiments (*n* = 4). Two-way ANOVA with Dunnett’s post hoc test was used to determine significance (adjusted *p*-value * *p* < 0.05, ** *p* < 0.01). ± SEM are displayed.

To assess the effects of the repeated-dose BPA exposure on the growth rate during the 3D neural induction, we measured the surface area of differentiating spheroids from brightfield images obtained throughout the induction (Figure 4A). From D3 to D15, there were no significant differences in the size of spheroids in each treated group in comparison to the vehicle-treated group. Remarkably, by D21 of the induction, significant decreases in spheroid size were observed; compared to the vehicle control group, the mean spheroid sizes were reduced by 32%, 25%, and 27% in 0.01, 0.1 and 1 µM BPA treated groups, respectively (Figure 4B).

### 3.4 Effects of BPA exposure on mitochondrial and ROS levels

**FIGURE 5:**
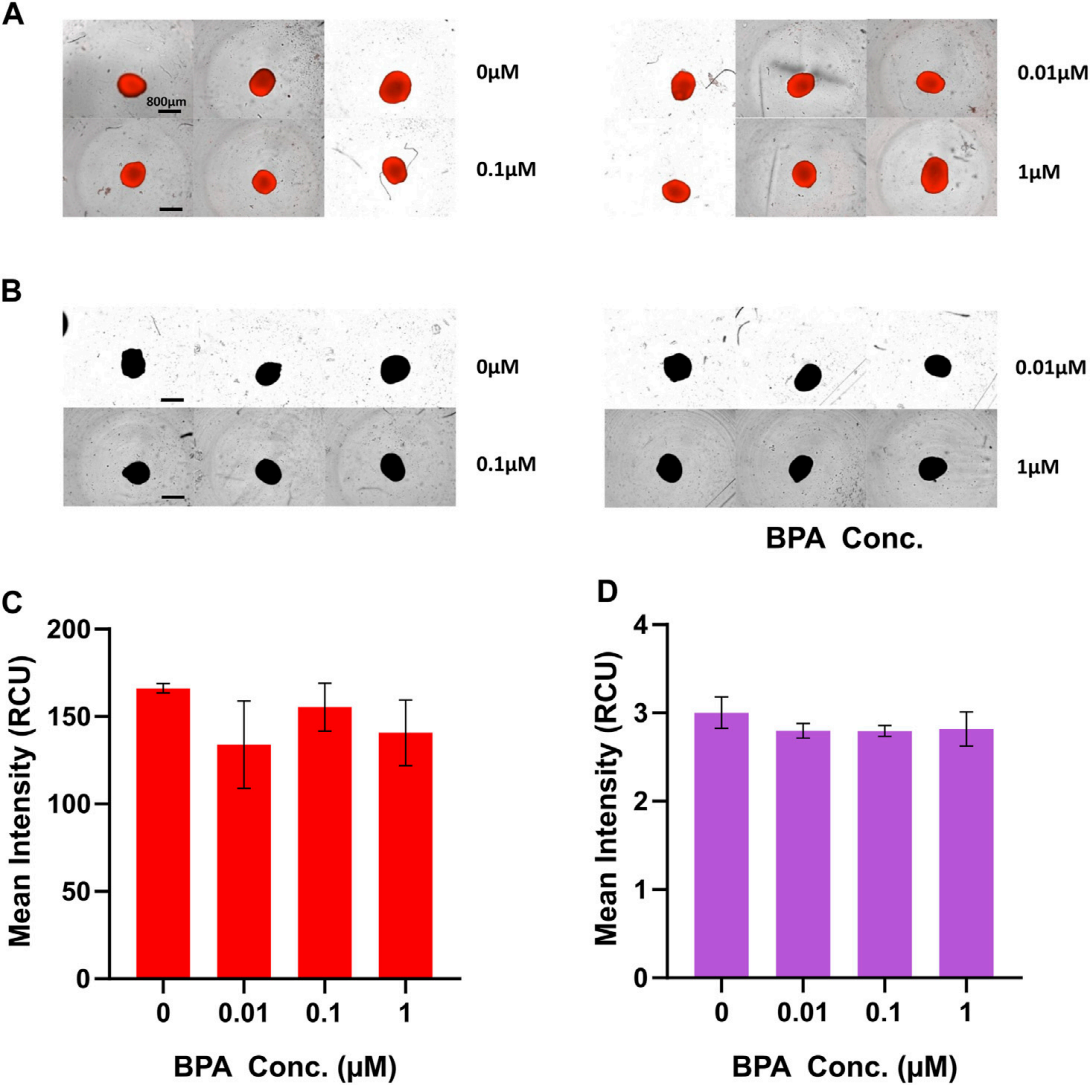
Fluorometric analysis of mitochondrial and ROS levels at D21 of the BPA-treated neural induction. Images of D21 spheroids captured by the IncuCyte^®^ Live Cell Analysis System for measurements of **(A)** mitochondrial and **(B)** ROS levels. Scale bar, 800 µm. **(C)** Mean MitoTracker Deep Red fluorescence intensity and **(D)** mean CellROX™ Deep Red fluorescence intensity of BPA treated spheroids at D21 quantified using the IncuCyte^®^ Live Cell Analysis System. 0 µM represents the vehicle-treated control groups. Three independent experiments were performed (*n* = 3). ± SEM is displayed. One-way ANOVA with Dunnett’s post hoc test was used to determine significance.

To investigate alterations of mitochondrial levels in BPA-treated D21 NSC spheroids, we used MitoTracker Deep Red fluorescent probes, commonly utilized for viable cell staining of mitochondria. There was no significant difference in mean fluorescence intensity in each treated group compared to vehicle-treated control at D21 (Figures 5A, C); suggesting that BPA treatment induced no long-term alterations to mitochondrial levels after 21 days of treatment. Similarly, we assessed whether BPA treatment affected cellular ROS levels using CellROX™ Deep Red fluorescent probes. We observed no significant change to mean fluorescence levels in BPA-treated groups at D21 (Figures 5B, D), indicating that BPA treatment at our experimental concentrations had no effect on cellular ROS levels.

### 3.5 Assessment of BPA-induced effects on NSC transcripts by RT-qPCR

**FIGURE 6:**
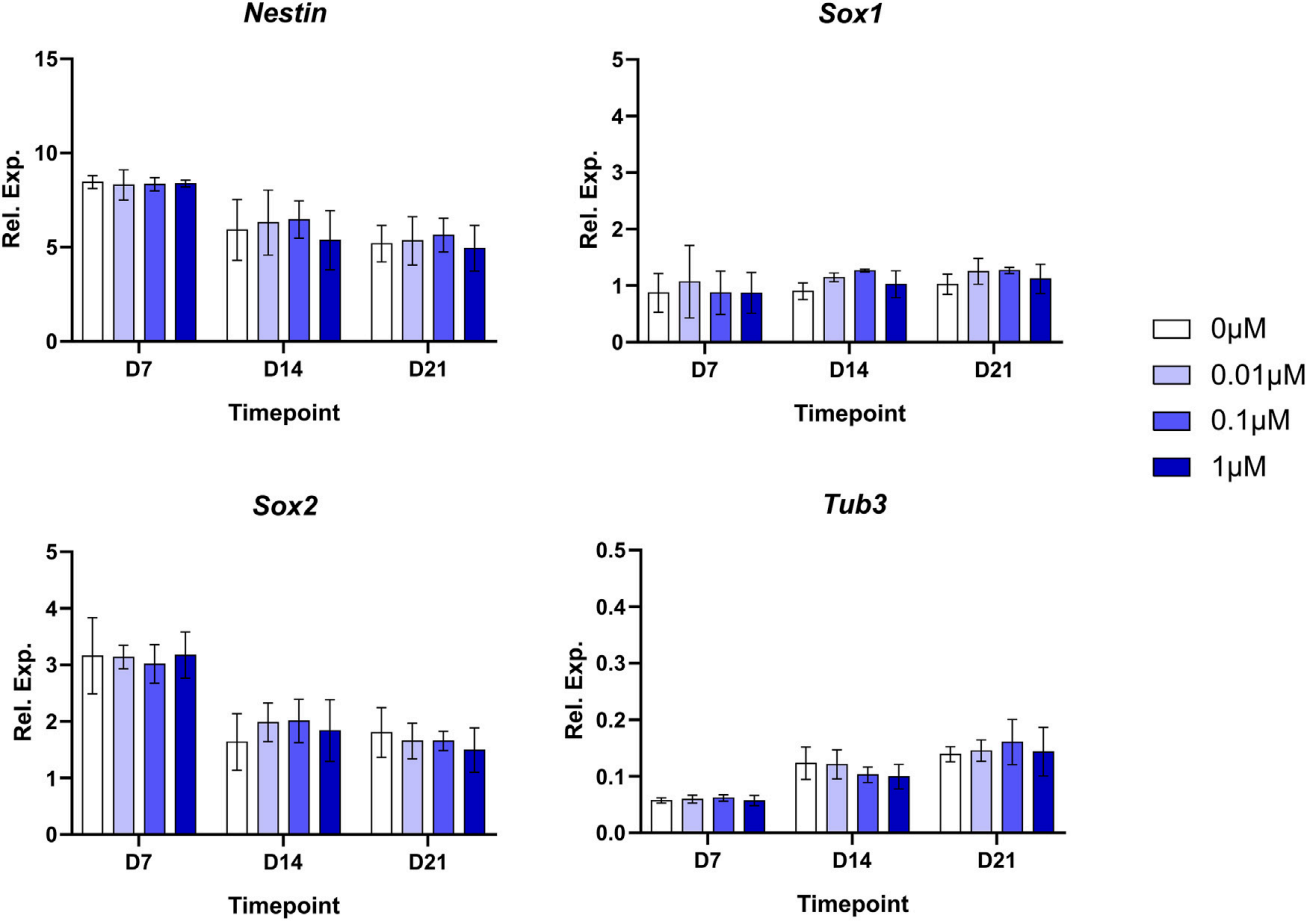
Weekly real-time qPCR measurements during the BPA-treated 3D neural induction. Graphs represent normalized relative expression values. GAPDH was used as a reference gene and data were normalized using Takara fetal brain RNA. Results shown are from 3 independent experiments (*n* = 3). Two-way ANOVA with Dunnett’s post hoc test was used to determine significance. ± SEM are displayed.

To investigate potential changes to key NSC transcripts arising due to BPA exposure, and to determine whether there were alterations to the rate of hiPSC to NSC differentiation, we performed a weekly investigation of gene expression using RT-qPCR during the neural induction (Figure 6). We measured the relative expression of key neuroectodermal markers Nestin and Sox1, as well as Sox2 and Tub3. No significant alterations in the transcript expression levels of these genes were detected at D7, D14 or D21 in response to BPA exposure throughout the neural induction, suggesting that there was no change to the rate of the differentiation compared to controls.

### 3.6 Proteome remodelling effects of BPA treatment on the neural induction

**FIGURE 7:**
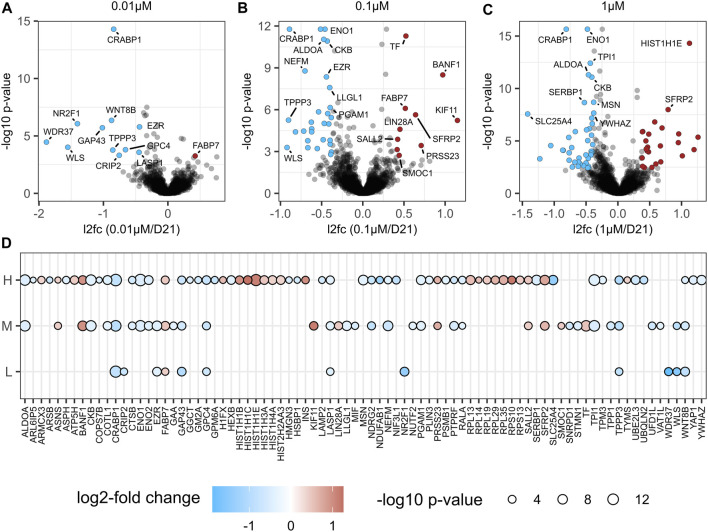
Proteome analysis after BPA treated Neural Induction. Volcano plot visualization of proteome alterations in NSCs treated with **(A)** 0.01 µM (*n* = 4), **(B)** 0.1 µM (*n* = 4) and **(C)** 1 µM (*n* = 4) BPA for 21 days during the neural induction. **(D)** Bubble plot of significantly altered proteins (adjusted *p*-value <0.05 and fold change >1.3) in at least one comparison are shown; L (0.01 µM/D21); M (0.1 µM/D21); H (1 µM/D21). The color of the circles corresponds to the log2 fold change of protein (blue downregulation, red upregulation) and the size of the circle correlates with Benjamini-Hochberg-adjusted–log10 *p*-value. L, low concentration; M, middle concentration; H, high concentration. 0 µM signifies the vehicle-treated control group.

To investigate the effect of BPA exposure on NSC proteome remodelling in an unbiased and comprehensive manner, we performed a label-free LC-MS/MS of NSCs treated with repeated doses of 0 µM (vehicle control, *n* = 4), 0.01 µM (*n* = 4), 0.1 µM (*n* = 4) and 1 µM (*n* = 4) BPA during the *in vitro* 3D neural induction (Figure 3C). Compared to the vehicle-treated control group, 11, 39 and 66 proteins were altered in abundance in 0.01, 0.1 and 1 µM BPA-treated groups, respectively (Figures 7A–D, Supplementary Tables S4D–G). Interestingly, several proteins were consistently changed in abundance in all BPA-treated groups, regardless of concentration. These alterations included decreases to canonical Wnt signalling protein Wnt-8b, and GAP43 (Neuromodulin), as well as GPC4, and TPPP3 (p20). Conversely, there was an increased abundance of FABP7 in all BPA-treated groups. No change was detected to the levels of key NSC proteins Nestin, Sox1, Sox2 and Tub3 in BPA-treated groups compared to the vehicle-treated control group at D21, in accordance with our RT-qPCR data (Figure 6). The observed alterations to the proteome profiles of BPA-treated NSCs demonstrate a dose-dependent increase in the quantity of differentially abundant proteins. Several of the proteins altered in abundance inclusively in all BPA-treated groups are involved in critical processes in NSC maintenance and fetal brain development.

### 3.7 Protein network mapping in BPA-treated NSCs

**FIGURE 8:**
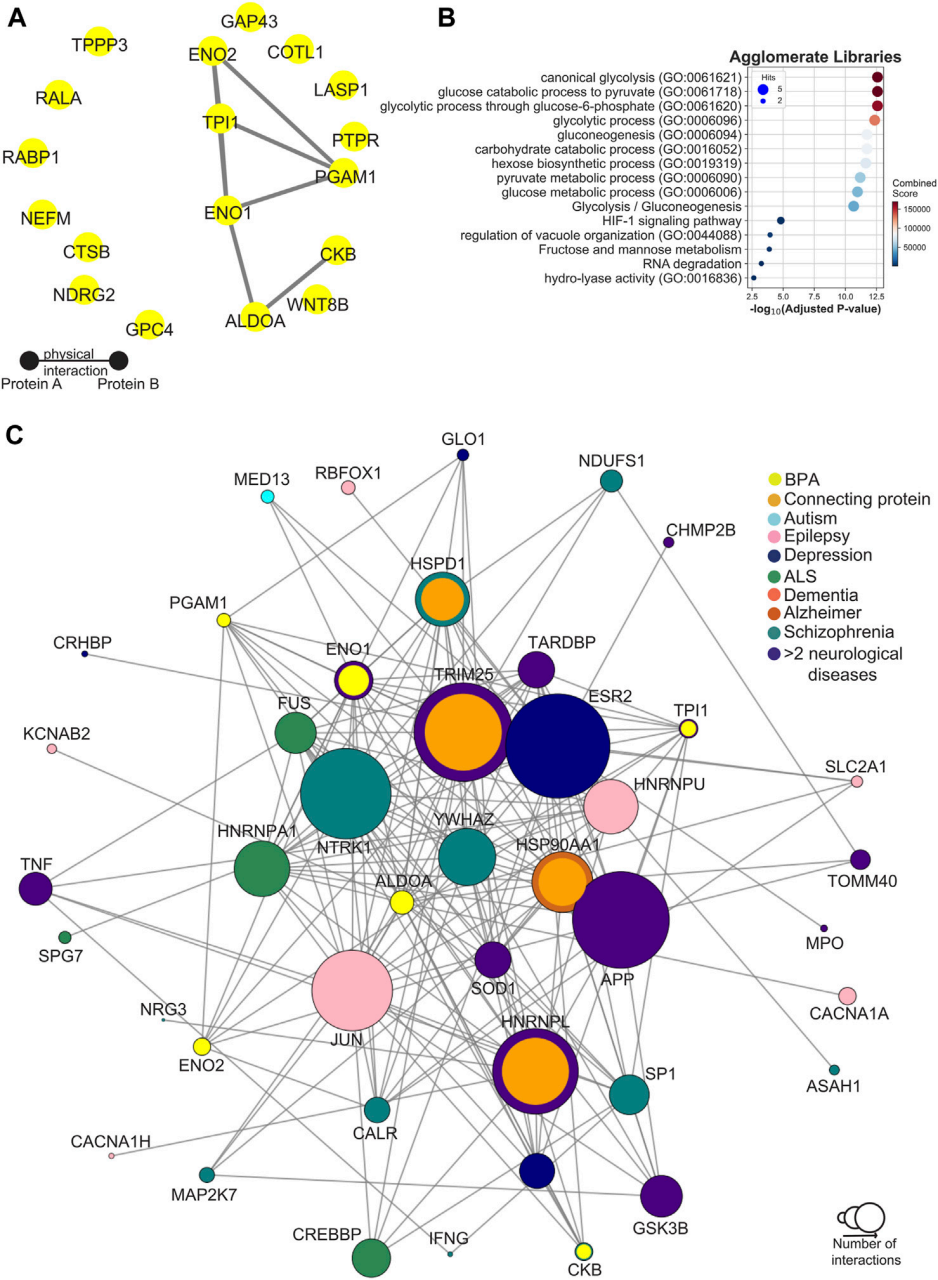
Network analysis of BPA-downregulated core proteins. **(A)** Protein-Protein Interaction (PPI) Network of the 18 downregulated proteins in both 0.1 and 1 µM BPA-treated groups. **(B)** BPA bubble plot of the top 15 enriched terms of the six BPA-downregulated core proteins. Bubbles are placed based on their -log10 *p*-value. Bubble size corresponds to the number of proteins that are found in each term, while their colour reflects the enrichment combined score **(C)** BPA downregulated core and disease-proteins network. Each node represents a protein, and its size is given by the global degree in the human PPI. The inner colour of each node represents whether that protein is one of the six BPA-downregulated core proteins (yellow), or a connecting protein (orange) or a disease-associated protein (colour-specific for each disease). If a protein is associated with more than 2 diseases, then the colour indigo will be used. The outlier color is used for BPA-downregulated core proteins and connecting proteins that have already been observed to be associated with one or more diseases.

To gain a better understanding of the molecular interactions and roles of the differentially abundant proteins in disease, we extensively mapped them in a human protein-protein interaction (PPI) network consisting of 18,816 proteins and 478,353 physical interactions (Menche et al., 2015; Alanis-Lobato et al., 2017; Luck et al., 2020). Overall, we observed that the number of differentially abundant proteins and their connectivity in the PPI increased with increasing levels of BPA exposure (0.01 µM BPA *p*-value:3e-5, 0.1 µM BPA *p*-value: 1e-09, 1 µM BPA *p*-value: 2e-26. Supplementary Figure S5A), indicating a growing number of proteins that contribute to the same molecular processes in a dose-dependent manner. Notably, this trend was more pronounced for the downregulated proteins (Supplementary Figure S5B). Due to the observed dose-dependency of the results, we focused on proteins that exhibited downregulation at both 0.1 and 1 µM BPA concentrations. Among the 18 downregulated proteins that met this criterion, we observed 6 proteins (ENO1, ENO2, TPI1, PGAM1, ALDOA, and CKB) tightly interacting in the PPI (*p*-value: 1.2e-12), which we define as BPA-downregulated core (Figure 8A) and that resulted to be enriched for the glycolytic pathway and the HIF-1 signalling pathway (Figure 8B; Supplementary Table S7B).

At this point, intrigued by the potential pathological phenotype of this dysregulated core, we investigated its representation on a list of 11,099 diseases compiled from DisGeNET (Piñero et al., 2015) calculating for each pairwise combination of their genetic overlap by computing a Jaccard Index (see methods), finding a strong association with “Enzymopathy” (FDR: 3.3e-05) and “Acute Schizophrenia” (FDR:0.001) (Supplementary Table S7E).

To incorporate network topological features on the comparisons between the BPA-downregulated core and the diseases retrieved from DisGeNET, we used network proximity (Guney et al., 2016), identifying several neurological disorders on the predicted list, such as “Alzheimer’s Disease” (FDR: 1e-06), “Amyotrophic Lateral Sclerosis” (FDR: 4e-07), and “Schizophreniform Disorders” (FDR: 4e-07) (Supplementary Table S7F).

To further understand the potential pathological implications of the BPA downregulated core proteins, we created a network that included these proteins and their shortest connections to a group of neurological disorders, including Schizophrenia, AD, Dementia, Depression, Autism behaviour, and ALS (Figure 8C). Most BPA-perturbed proteins interact with hubs in the brain known to be involved in several neurological disorders, such as APP (Sun et al., 2008; Jakobsson et al., 2013) and ESR2 (Pinsonneault et al., 2013).

We observed that the observed changes in ALDOA and ENO1 expression could induce various neurological disorders by modulating their direct interactors. For example, ALDOA interacts with both HDAC, and GLO1, which have been previously associated with depression (Tsankova et al., 2006; McMurray et al., 2014; 2018). Similarly, ALDOA also interacts with SOD1, and FUS two of the few known causal genes in ALS (Miller et al., 2013; Deng et al., 2014).

To investigate this further, we analysed the transcriptomic expression of those genes that by interacting with ENO1 and ALDOA could lead to ALS in the largest ALS cohort to date (Humphrey, 2022). We found that their expression was significantly lower in ALS patients compared to controls in the cervical and lumbar regions (Supplementary Tables S6A, B).

In summary, these results suggest that BPA may have a dose-dependent effect on neurodevelopment by affecting not only the number of differentially expressed proteins, but also their interactions, potentially contributing to the impairment of the glycolytic metabolism, which is known to be altered in various neurological conditions.

## 4 Discussion

In recent years, several groups have investigated BPA-induced aberrations to the proliferative capacity of NSCs and NPCs, with conflicting results depending on BPA concentration and exposure duration. Studies using rat and human NSCs *in vitro* have demonstrated that BPA increased NSC proliferation and induced aberrant differentiation (Dong et al., 2021; Gill and Kumara, 2021). In contrast, studies have also shown that BPA exposure causes decreased proliferation in mouse NSCs (Kim et al., 2007), impaired the proliferation of rat NSCs *in vitro* and *in vivo* (Tiwari et al., 2015; Agarwal et al., 2016), and human NSCs from umbilical cord blood (Huang et al., 2019). Additionally, studies have described BPA-induced impairments to NSC differentiation, for example, inhibited NSC differentiation (Agarwal et al., 2016; Huang et al., 2019; Dong et al., 2021) and conversely, premature neurogenesis (Kinch et al., 2015). These contrasting results may be attributed to the non-monotonic properties shared by BPA and other endocrine-disrupting compounds, rendering it difficult to reach a consensus regarding BPA effects on neural development across various experimental conditions (Vandenberg, 2014; Villar-Pazos et al., 2017). The effects of BPA may also diverge depending on the specific developmental timing of exposure. Notably, previous *in vitro* studies have focused on the effects of BPA on established NSC populations and their differentiation, overlooking the earlier differential processes of embryonic brain development, from which NSCs are derived.

To date, there remains a lack of human relevant data on BPA exposure during brain development, as most currently available information was obtained from animal studies. In our study, we showed that our 3D *in vitro* system was an effective and stable model for hiPSC to NSC differentiation. Using RT-qPCR and ICC visualization, we found increases in the expression of typical neuroectodermal and NSC markers, such as Nestin and Sox2, throughout the *in vitro* 3D neural induction on a protein and mRNA level. Furthermore, our proteomics study complemented this data, and consolidated the robustness of our model by demonstrating increases to a considerable number of key neural lineage and NSC markers by D21 (Červenka et al., 2021; Galiakberova and Dashinimaev, 2020; Shin et al., 2007; Yun et al., 2012). Due to its human origin, this model, therefore, provides a new approach method (NAM), suitable for generating human relevant, highly translatable data for toxicity during the early stages of embryonic brain development. Despite the beneficial 3D microenvironment provided by spheroids, they are only comprised of a single cell type, in our case NSCs, and do not entirely reflect the intricate tissue structure of the developing brain (Augustyniak et al., 2019). Future studies investigating the effects of BPA on NSC development could therefore also utilize brain organoids, which closely replicate brain developmental processes. Organoids have a highly organized architecture and are comprised of several cell types, demonstrating considerable promise for toxicological study which can complement data regarding BPA effects obtained using NSC spheroids (Lancaster and Knoblich, 2014; Caipa Garcia et al., 2022).

Here, we examined the effects of nano to micromolar concentrations of BPA (0.01 µM–1 µM) that are aligned with environmentally relevant concentration levels (Ribeiro et al., 2017; 2019; Zahra et al., 2022). This concentration range allowed us to model, as closely as possible, the effects of a real-life, repeated environmental exposure to BPA in an *in vitro* setting. Performing a repeated-dose exposure during the 3D neural induction over 21 days encompassed the duration of the differentiation of hiPSCs to NSCs (Chambers et al., 2009; Galiakberova and Dashinimaev, 2020) and facilitated the investigation of the effects of BPA exposure on the *in vitro* neural induction of hiPSCs for the first time.

Our results demonstrated that cell viability levels were not affected after 14 or 21 days of the BPA-treated neural induction. This is in line with other *in vitro* studies that reported no change to the viability of neural stem and progenitor cells within the same concentration range of BPA, albeit after periods of acute exposure (Kim et al., 2007; Tiwari et al., 2015). The present study also showed a reduction in the spheroid size in all BPA-treated groups at D21, suggesting BPA exerted inhibitory effects on the proliferation of NSCs. These observations support current literature that demonstrated inhibited NSC/NPC proliferation upon BPA exposure (Kim et al., 2007; Tiwari et al., 2015; Huang et al., 2019; Rebolledo-Solleiro et al., 2021). Taken together, the reduced spheroid size in BPA-treated groups without alterations to cellular viability at D21 implied that molecular mechanisms were responsible for the observed changes.

Both *in vitro* and *in vivo* studies have demonstrated that BPA exposure can affect mitochondrial dynamics and cellular ROS levels in NSCs/NPCs, especially at higher concentrations (Kim et al., 2007; Agarwal et al., 2016; Kobayashi et al., 2020; Wang et al., 2021). In our study, we did not observe any significant alterations to cellular ROS and mitochondrial levels from a qualitative assessment at D21 of the BPA-treated neural induction in 0.01, 0.1, or 1 µM treated groups. However, due to light scatter and inadequate label penetrance into samples, end-point fluorescence-based imaging of fixed spheroids is mostly limited to the peripheral layers of cells, while it is also a possibility that short-term changes occurred at timepoints prior to D21 of the differentiation. Also, the quantification of ROS levels *in vitro* remains challenging, especially when considering that their abundance is continuously altered by various chemical reactions, diffusion, and compensatory mechanisms (Murphy et al., 2022).

Acute BPA exposure of established NSCs at concentrations of 1 µM has been demonstrated to induce decreases to MAP2 and GFAP, markers of neuronal and glial specification respectively, as well as increases to NSC maintenance markers Nestin and Sox2 (Dong et al., 2021). Taking this into account along with previously discussed evidence that BPA perturbed NSC differentiation and neurogenesis, we investigated whether BPA affected the differentiation rate of hiPSCs to NSCs. In our study, no alterations were detected to the mRNA or protein levels of neuroectodermal markers Nestin and Sox1. Additionally, we detected no changes to the mRNA levels of Sox2, a marker of NSC multipotency in non-committed NSCs (Shimozaki, 2014), or Tub3, a typical marker of immature neurons that could indicate neuronal differentiation (Katsetos et al., 2003), suggesting there was no alteration to the rate of hiPSC to NSC differentiation.

To date, several studies have reported BPA-induced aberrations to vital molecular pathways that are important for proper NSC maintenance and neural differentiation. *In vivo* and *in vitro* rat models have shown that BPA disrupts the canonical Wnt signalling pathway, which has a critical influence on NSC/NPC proliferation and differentiation, as well as the pathophysiology of certain NDDs (Fang et al., 2015; Tiwari et al., 2015; Mulligan and Cheyette, 2016; Tiwari et al., 2016; Üstündağ and Emekli-Alturfan, 2020). Moreover, Dong et al., 2021 reported that a 1 µM BPA treatment of human NSCs *in vitro* led to the modulation of ERRα, TGF-β and p53 signalling pathways, which maintain roles in the stemness maintenance of NSCs. However, the debate surrounding the molecular effects and consequences of BPA exposure on the developing fetal brain, and its associations to brain disorders, is ongoing.

To investigate the molecular perturbations to NSCs derived from the BPA-treated neural induction, we investigated proteomic alterations in BPA-treated groups. Proteomics analysis revealed several key proteins with functions related to the maintenance of NSC proliferation and differentiation that were significantly altered inclusively in 0.01, 0.1 and 1 µM-treated groups. We detected a decrease to Wnt-8b of the canonical Wnt signalling pathway in all treated groups. Interestingly, Wnt-8b is expressed in the neural tube, while it also plays a role in neuroectodermal patterning (Kim et al., 2002; Ciani and Salinas, 2005). A BPA-induced decrease to Wnt-8b could therefore affect the regional patterning specification of neural stem cells during development. Our findings of reduced Wnt-8b levels and spheroid size in all BPA-treated groups are supported by previous rat model studies that uncovered BPA-induced suppression of NSC proliferation due to aberrations to the canonical Wnt signalling pathway (Tiwari et al., 2015; Tiwari et al., 2016), and furthermore, for the first time, we confirmed that BPA can induce canonical Wnt alterations in a human NSC model.

In addition, we observed a decrease to Neuromodulin (GAP43) in all BPA-treated groups, a nervous system-specific protein required in mitotic neural progenitors (Brittis et al., 1995; Esdar et al., 1999). GAP43 is uniformly expressed in proliferating areas of the developing embryonic brain, while a lack of GAP43 expression results in suppressed neural progenitor proliferation (Kanazir et al., 1996; Mani et al., 2001; Mishra et al., 2008). Interestingly, the absence of GAP43 expression induces aberrations to the differentiation and neural/glial commitment of multipotent precursor cells (Mani et al., 2001; Shen et al., 2004; 2008). Additionally, TPPP3 (p20) was decreased in all BPA-treated groups. TPPP3 is a cyclin-dependent kinase inhibitor and a member of the TPPP family, robust modulators of proliferation in most developmental processes (Oláh et al., 2017; Shukla et al., 2018). Knockdown/inhibition of TPPP3 has resulted in decreased proliferation and cell cycle arrest in various human and mouse tumour cell lines (Li et al., 2016; Oláh et al., 2022), suggesting it may play a role in the observed alterations to NSC proliferation.

Another protein decreased in all BPA-treated groups was Glypican-4 (GPC4), expressed by multipotent NSCs in the ventricular zone of the developing CNS. Upon NSC commitment, GPC4 expression desists (Hagihara et al., 2000). GPC4 depletion has also been demonstrated to affect the proliferation and maintenance of NSCs. Notably, GPC4 downregulation in NSCs tips the balance from maintaining the NSC pool in favour of intermediate progenitors (Fico et al., 2012), leading to the premature depletion of NSCs. Intriguingly, it has also been shown that the modulation of stem cell fate by GPC4 is primarily limited to the positive regulation of canonical Wnt signalling (Fico et al., 2012). Thus, the observed decreases to GPC4 in the present study provides a further mechanistic explanation to complement previous studies that implicated BPA-induced Wnt alterations in the suppression of NSC proliferation (Tiwari et al., 2015; Tiwari et al., 2016). Conversely, we observed an increase to FABP7 in all BPA-treated groups. FABP7 is expressed in neural stem/progenitors and during neuronal differentiation (Knobloch, 2017). FABP7 expression has been demonstrated to culminate during the transition of NSCs to radial glial progenitors and plays a role in their maintenance (Arai et al., 2005; Matsumata et al., 2016). Therefore, the increase of FABP7 in all treated groups could indicate that BPA-treated NSCs are at an advanced stage toward radial-glial progenitor transition.

Taken together, we uncovered BPA-induced changes to the levels of several proteins with important functional roles in NSC maintenance; supporting current literature and further elucidating potential molecular mechanisms implicated in the altered proliferation and differentiation of BPA-treated NSCs. The observed protein alterations could suggest that BPA-treated NSCs are at a more advanced differential stage in line with neural progenitors, which have a lower proliferative capacity. Therefore, it could be interesting for further study to distinguish whether BPA treatment precociously steers NSCs to a more ‘primed’ NPC state for neurogenesis, which could aid in the explanation of decreased proliferation and premature depletion of the NSC pool. A current caveat regarding the use of mass spectrometry-based proteomics is that peptides in low abundance may be under-sampled or altogether undetected due to dynamic range limitations, even when using cutting-edge mass spectrometers. Since proteins in low abundance, such as transcription factors, are often underrepresented in proteomic investigations, it remains a possibility that other important BPA-induced proteome alterations were undetected with this method. Future advancements to the sensitivity of mass spectrometry technology may enable issues with peptide abundances to be addressed, which would allow for more comprehensive investigations into the effects of BPA on the whole NSC proteome (Fonslow et al., 2011; Ding et al., 2013; Timp and Timp, 2020).

Our results demonstrated that BPA exposure at 0.1 and 1 µM potentially contributed to the impairment of the glycolytic pathway. Glycolysis is a critical metabolic process underlying NSC proliferation, and interestingly, several studies have demonstrated that glycolysis, rather than oxidative phosphorylation (OXPHOS), is the primary source of ATP synthesis in NSCs (Zheng et al., 2016; Iwata and Vanderhaeghen, 2021). The process of neurogenesis includes the proliferation of NSCs to NPCs, followed by neuronal differentiation. This is coupled with a progressive metabolic shift; as cells differentiate from NSC stage towards neurons, the predominant process for ATP synthesis transitions from glycolysis to OXPHOS (Zheng et al., 2016; Maffezzini et al., 2020; Iwata and Vanderhaeghen, 2021). Decreases to proteins of the glycolytic pathway could therefore also support the argument that BPA-treated NSCs are at a more advanced NPC stage as they begin to decrease glycolysis and advance their differential status. However, whether BPA-induced impairments to proliferation are a cause or a consequence of decreases to proteins of the glycolytic pathway is currently unclear.

Evidence from the present study and previous reports may explain the BPA-induced mechanisms leading to altered expression of proteins of the glycolytic pathway. BPA is known to bind to estrogen receptors (ERs), which are ubiquitously located, ligand-activated transcription factors. ERs modulate estrogen-dependent signalling pathways involved with the regulation of cell proliferation and differentiation in several cell types and tissues, including NSCs in the brain (Bustamante-Barrientos et al., 2021). Interestingly, ERs are known to exert their intracellular effects via PI3k/ATK signalling, an intracellular signalling pathway responsible for promoting proliferation, growth, and metabolism in response to extracellular stimuli (MacKay and Abizaid, 2018; Hoxhaj and Manning, 2020). The PI3K-Akt signalling pathway also maintains a critical role in the stimulation of glycolysis (Xie et al., 2019). A key downstream effector protein of this pathway includes HIF-1α of the HIF-1 signalling pathway. Although the oxygen-dependent regulation of HIF-1α is strongly associated with negative regulation by prolyl hydroxylases, HIF-1α is also modulated via PI3K/Akt signalling in non-hypoxic conditions (Masoud and Li, 2015). HIF-1 signalling plays a critical role in the regulation of metabolic enzymes and transporters that are responsible for promoting glycolysis (Kim et al., 2014; Del Rey et al., 2017). We observed decreases to proteins involved in the HIF-1 signalling pathway in 0.1 and 1 µM BPA-treated groups. Moreover, p53, another downstream effector of PI3K/Akt signalling, is involved in glycolytic suppression (Kim et al., 2014), and aberrations to p53 signalling have been previously reported in human NSCs after 1 µM BPA treatment (Dong et al., 2021). An exciting avenue for further research could therefore be the investigation of the metabolic status of BPA-treated NSCs and neurons. This could further uncover the role of BPA-induced glycolytic aberrations in the altered proliferation and differentiation reported after BPA treatment during neural developmental processes, which until now is relatively unexplored.

Many studies have pointed to the close connection between glycolytic brain metabolism, NDDs and NDs (Nascimento and Martins-de-Souza, 2015; Zhang et al., 2021; Li and Sheng, 2022). Metabolic gradients are fundamental factors in the regulation of NSC fate (Angelopoulos et al., 2022), and neural tube defects have been associated with the faulty regulation of glucose metabolism during pregnancy (Paddock et al., 2017; Keuls et al., 2020). Additionally, decreased glucose and oxygenation rates are linked to ageing and NDs (Camandola and Mattson, 2017), and to impairments of synaptogenesis during embryo development (Goyal et al., 2014).

Here, we show that BPA decreases the protein expression of members of the glycolytic pathway in NSCs, potentially leading to neurological defects that are not limited to the gestational period, and that could lead to NDDs and NDs. For instance, we showed that ENO1 and ALDOA interact with MED13, which has been associated with a broad range of NDDs including encephalopathy, intellectual disability, and autism (De Nardi et al., 2021; Trivisano et al., 2022). On the other side of the spectrum, we observed that glycolytic impairments could contribute to the onset of ALS through the interaction of ALDOA with SOD1, FUS, and ESR2. Interestingly, the decreased expression of ESR2, APP, and SPG7, direct interactors of ALDOA and ENO1 of the glycolytic pathway, has been confirmed in the cervical and lumbar regions of an independent cohort of ALS patients. Although there is currently a lack of evidence that directly connects BPA exposure to ALS, it remains a possibility that other physiological or metabolic disturbances induced by BPA, such as glycolytic defects, could play a role in ALS development. For instance, several pre-clinical studies suggest that altered CNS glucose metabolism and transport could lead to ALS progression (Tefera et al., 2021), and emerging reports from mouse model studies have demonstrated glycolytic impairments in ALS (Ferraiuolo et al., 2011; Tefera and Borges, 2019; Tefera et al., 2019). To summarize, we show that BPA exposure could lead to fundamental metabolic changes in the developing brain that could impair several critical developmental processes from neural tube formation to synaptogenesis, increasing the risk to develop several neurological disorders at any age in life.

In conclusion, we present a novel NAM for the investigation of toxic exposures on NSC differentiation during the earliest stages of CNS development. We demonstrated that a repeated-dose exposure over 21 days with sub-cytotoxic, environmentally relevant BPA concentrations decreased spheroid size during the neural induction of hiPSCs to NSCs, thus supporting previous literature showing that BPA inhibits the critical NSC property of proliferation. We identified BPA-induced alterations to several proteins with important functions in NSC proliferation and differentiation, and the glycolytic pathway, with implications for our understanding of the effects of BPA exposure on NSC maintenance and differentiation during embryonic brain development. In-line with the DOHaD concept, we provide further mechanistic insight into the links between BPA exposure during CNS development and several brain disorders, suggesting a role for BPA-induced changes to the glycolytic pathway in the pathophysiology of NDDs such as intellectual disability and ASD, as well as NDs including ALS.

## Data Availability

The datasets presented in this study can be found in online repositories. The names of the repository/repositories and accession number(s) can be found in the article/Supplementary Material.
