# Supplementary material for: Proteomics and disease network associations evaluation of environmentally relevant Bisphenol A concentrations in a human 3D neural stem cell model
Source: Front Cell Dev Biol. 2023 Aug 16;11:1236243. doi: 10.3389/fcell.2023.1236243 (PMC10472293; doi:10.3389/fcell.2023.1236243)
Supplement: Supplementary file 2 [file Table1.docx]

Supplementary Table S1 - Antibody List

| **Manufacturer/ Cat. No.** | **Host/ Clonality** | **Target** | **Isotype** | **Dilution** |
| --- | --- | --- | --- | --- |
| Sigma-Aldrich/ MAB5326 | Mouse/ Monoclonal | Nestin | IgG | 1:200 |
| R&D systems/ AF3369 | Goat/ Polyclonal | Sox1 | IgG | 1:100 |
| Sant Cruz/ sc365823 | Mouse/ Monoclonal | Sox2 | IgG1 | 1:100 |
| Cell Signal/ 5741s | Rabbit/ Monoclonal | Vimentin | IgG | 1:100 |
