# Supplementary material for: Proteomics and disease network associations evaluation of environmentally relevant Bisphenol A concentrations in a human 3D neural stem cell model
Source: Front Cell Dev Biol. 2023 Aug 16;11:1236243. doi: 10.3389/fcell.2023.1236243 (PMC10472293; doi:10.3389/fcell.2023.1236243)
Supplement: Supplementary file 3 [file Presentation1.pptx]

## Slide 1
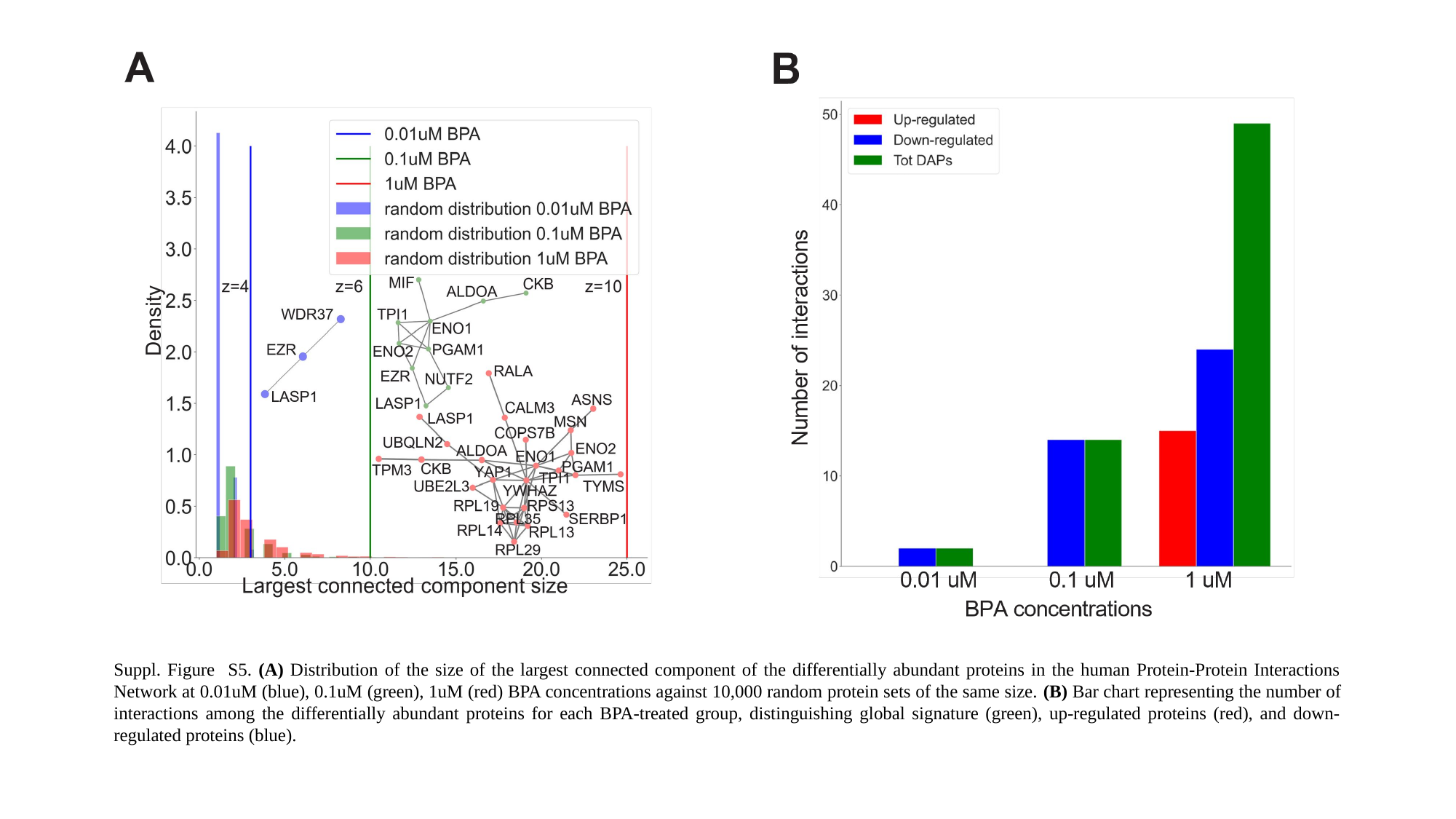

Suppl. Figure S5. (A) Distribution of the size of the largest connected component of the differentially abundant proteins in the human Protein-Protein Interactions Network at 0.01uM (blue), 0.1uM (green), 1uM (red) BPA concentrations against 10,000 random protein sets of the same size. (B) Bar chart representing the number of interactions among the differentially abundant proteins for each BPA-treated group, distinguishing global signature (green), up-regulated proteins (red), and down-regulated proteins (blue).
