# Supplementary material for: Proteomics and disease network associations evaluation of environmentally relevant Bisphenol A concentrations in a human 3D neural stem cell model
Source: Front Cell Dev Biol. 2023 Aug 16;11:1236243. doi: 10.3389/fcell.2023.1236243 (PMC10472293; doi:10.3389/fcell.2023.1236243)
Supplement: Supplementary file 4 [file Table2.docx]

| ***GAPDH*** | Fwd: CTCTCTGCTCCTCCTGTTCGAC | Rv: TGAGCGATGTGGCTCGGCT |
| --- | --- | --- |
| ***Vimentin*** | Fwd: GACCAGCTAACCAACGACAAAG | Rv: CGCATTGTCAACATCCTGTCTG |
| ***Sox1*** | Fwd: TAGTAAGGCAGGTCCAAGCA | Rv: GGGTGGTGGTGGTAATCTCT |
| ***Sox2*** | Fwd: ACCAGCTCGCAGACCTACA | Rv: TCGGACTTGACCACCGAAC |
| ***Nestin*** | Fwd: ACTGAAGTCTGCGGGACAAG | Rv: CAGTGGTGCTTGAGTTTCTG |
| ***Oct-4*** | Fwd: AAAGCGAACCAGTATCGAGAAC | Rv: GCCGGTTACAGAACCACACT |
| ***Pax6*** | Fwd: GCCAGCAACACACCTAGTCA | Rv: TGTGAGGGCTGTGTCTGTTC |
| ***Tubulin-3*** | Fwd: AACGAGGCCTCTTCTCACAA | Rv: GGCCTGAAGAGATGTCCAAA |

Supplementary table S2 - Primer sequences
