# Supplementary material for: Proteomics and disease network associations evaluation of environmentally relevant Bisphenol A concentrations in a human 3D neural stem cell model
Source: Front Cell Dev Biol. 2023 Aug 16;11:1236243. doi: 10.3389/fcell.2023.1236243 (PMC10472293; doi:10.3389/fcell.2023.1236243)
Supplement: Supplementary file 5 [file DataSheet1.pdf]

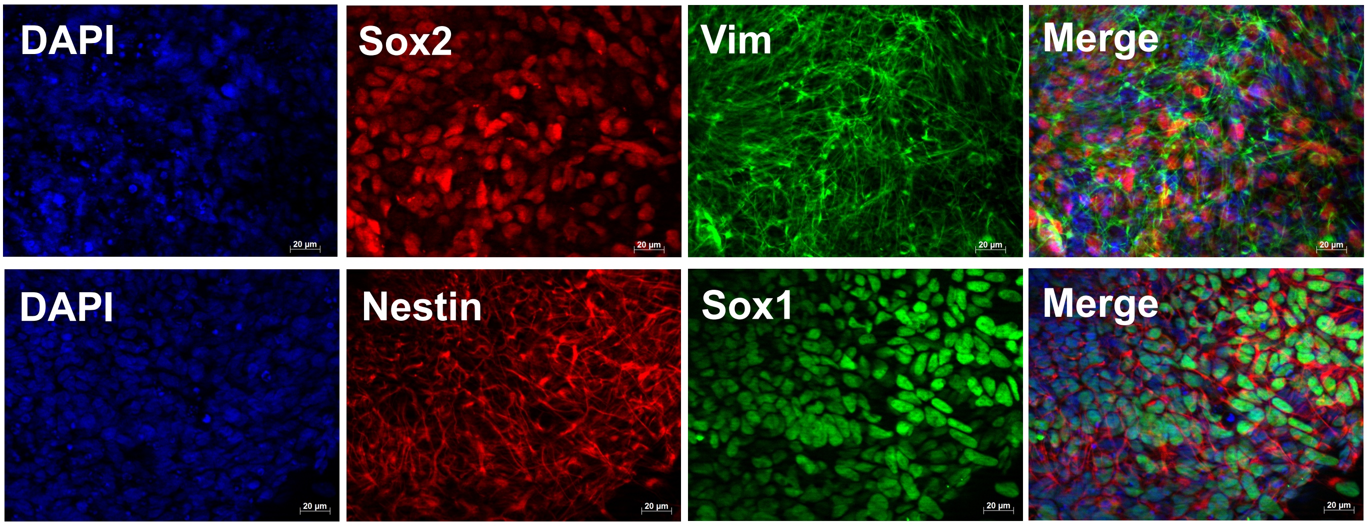

Suppl. Figure S3. Immunocytochemical detection of spheroids at D21 of the in vitro neural induction of hiPSCs. Neuroectodermal and neural stem cell markers were stained in 3D culture (scale bars displayed). The used fluorophores were Alexa 488 (green) and Alexa 594 (red). Nuclei were counterstained with DAPI (blue). Scale Bar, 20um.
