# Supplementary material for: Proteomics and disease network associations evaluation of environmentally relevant Bisphenol A concentrations in a human 3D neural stem cell model
Source: Front Cell Dev Biol. 2023 Aug 16;11:1236243. doi: 10.3389/fcell.2023.1236243 (PMC10472293; doi:10.3389/fcell.2023.1236243)
Supplement: Supplementary file 7 [file Presentation2.pptx]

## Slide 1
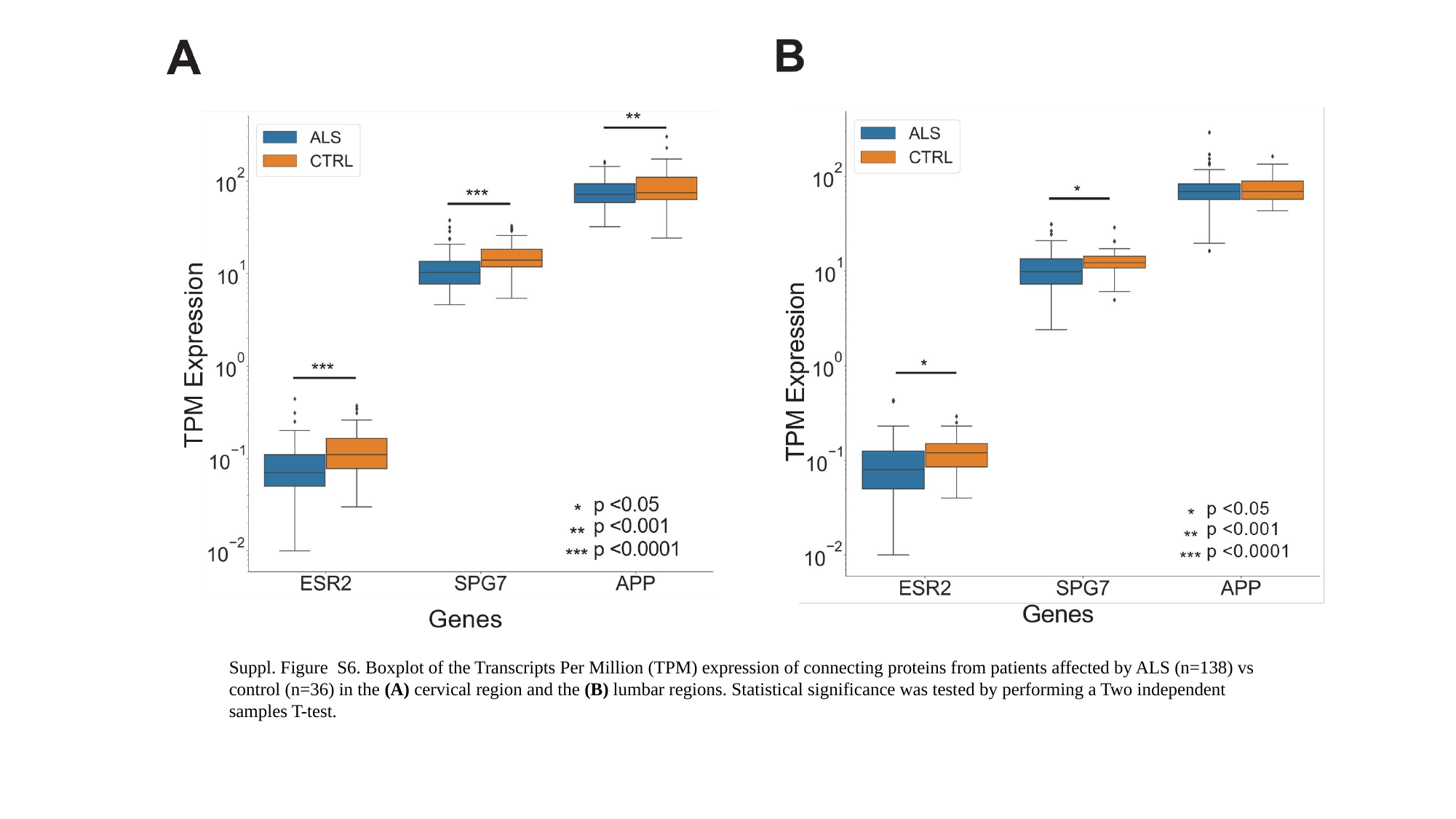

Suppl. Figure S6. Boxplot of the Transcripts Per Million (TPM) expression of connecting proteins from patients affected by ALS (n=138) vs control (n=36) in the (A) cervical region and the (B) lumbar regions. Statistical significance was tested by performing a Two independent samples T-test.
